# Cross-jurisdictional access to innovative drugs and devices: a five-year policy and practice review of the Hong Kong and Macao medicine and equipment connect in the Greater Bay Area of China

**DOI:** 10.3389/fmed.2026.1897761

**Published:** 2026-08-10

**Authors:** Bingxin Peng, Zhenjie Mo, Yan Yuan, Hui Yao, Liangkui Xu

**Affiliations:** 1 School of Pharmacy, Guangdong Food and Drug Vocational College, Guangzhou, China; 2 School of Chinese Materia Medica, Guangdong Pharmaceutical University, Guangzhou, China; 3 Department of Pharmacy, The First People's Hospital of Foshan, Foshan, China; 4 Department of Clinical Pharmacy, The Second People’s Hospital of Foshan, Foshan, China; 5 School of Pharmaceutical Engineering, Guangdong Food and Drug Vocational College, Guangzhou, China

**Keywords:** cross-jurisdictional drug access, delegated regulatory governance, expedited drug approval pathway, Guangdong-Hong Kong-Macao Greater Bay Area, Hong Kong and Macao medicine and equipment connect, pharmaceutical regulation, real-world evidence

## Abstract

Launched in 2020, the “Hong Kong and Macao Medicine and Equipment Connect” (HKMEC) policy introduced a novel delegated-authority model in China’s Greater Bay Area (GBA), enabling designated hospitals to use Hong Kong- or Macao-approved drugs and devices through Guangdong provincial approval, thereby bypassing standard National Medical Products Administration (NMPA) registration. To synthesize five-year implementation evidence, this review followed PRISMA-ScR guidelines and systematically searched Chinese (CNKI, Wanfang, VIP) and English (PubMed, Embase, ScienceDirect) databases covering 2020 to May 2026, supplemented by official documents. By early 2026, the in-use catalog comprised 115 products (45 drugs and 70 devices), while cumulative approvals reached 140 products across 71 institutions, serving more than 17,000 documented patient encounters (as of December 2025; Guangdong Provincial Health Commission, 8 December 2025), mainly in the fields of oncology, rare diseases, and chronic disease. While reliance on external approvals accelerated access, challenges remain, including fragmented real-world data (RWD) infrastructure, self-pay barriers, institutional disparities, and limited cross-border integration. Overall, HKMEC demonstrates a pragmatic delegated regulatory governance model with implications for subnational and transnational pathways, although three priority tasks remain: establishing a regulation-grade RWD platform, advancing financing reforms beyond self-pay, and pursuing substantive collaboration with Hong Kong and Macao. Sustained evidence-driven iteration will be essential to transform this pilot into a robust, scalable model bridging accelerated access and formal regulatory approval.

## 1 Introduction

The Guangdong-Hong Kong-Macao Greater Bay Area presents a regulatory divergence that few comparable mega-urban clusters face. Nine Guangdong municipalities, governed under mainland Chinese law, share a border with two Special Administrative Regions that operate under separate legal systems, customs territories, and regulatory frameworks shaped by the “one country, two systems” arrangement (1). The labor market and healthcare demand across this border are increasingly integrated, a trend reflected in growing cross-border patient flows. The practical consequence is significant: a resident of Guangdong who receives a diagnosis amenable to treatment with a drug licensed in Hong Kong but not yet approved by the NMPA has, until recently, faced a clinically urgent and logistically demanding choice: cross-border medical travel or forgoing the therapy (2).

The scale of this regulatory mismatch is substantial. Hong Kong’s Pharmacy and Poisons Board and the Macao Health Bureau collectively maintain access to a drug portfolio that leads the mainland registry by a clinically meaningful margin across oncology, rare diseases, and several chronic-disease categories (3, 4). This gap is not a consequence of regulatory negligence on either side; rather, it reflects the inherent sequencing of global registration filings by pharmaceutical companies and the finite throughput of China’s own approval pipeline (5–8).

The HKMEC policy was conceived as a targeted bridge across this regulatory gap, operating within the unique “one country, two systems” framework of the GBA (1). Its legal foundation rests on State Council authorization empowering the Guangdong provincial government to exercise approval authority normally reserved for the NMPA—a rare example of delegated regulatory governance in Chinese pharmaceutical regulation (9). The pilot began modestly, with a single institution, the University of Hong Kong–Shenzhen Hospital, designated to implement the scheme, reflecting a cautious and institution-based rollout strategy (2, 10). The initial mandate was explicitly time-limited, with the pilot program scheduled to expire in July 2021. That it has since expanded to 71 designated sites, over 140 approved products (cumulative figure as of December 2025), and more than 17,000 documented patient encounters (as of December 2025; Guangdong Provincial Health Commission, 8 December 2025)^1^ shows both genuine clinical demand and administrative commitment, albeit accompanied by operational challenges inherent to such regulatory innovation (11). Beyond its regulatory novelty, the policy carries substantive public-health significance: by operationalizing the “one country, two systems” framework within the health domain, it functions as a concrete mechanism for advancing health equity, strengthening rare-disease care, and promoting regional integration across the GBA.

Academic engagement with HKMEC has grown substantially since 2021, spanning public-policy implementation theory (12, 13), RWD quality governance (14), drug selection methodology (15), health technology assessment frameworks (16), regulatory-comparative analysis and administrative law scholarship. Despite this growing body of Chinese-language literature, no systematic English-language synthesis has emerged. This review aims to fill that gap by integrating evidence across these domains and situating the program within the broader international literature on expedited medicine access pathways (including the EU Conditional Marketing Authorization, the FDA Accelerated Approval designation, and Japan’s Sakigake priority review) to provide contextual reference for readers outside China’s regulatory environment (17–19).

This review seeks to answer three interrelated questions: How has the HKMEC program been designed and implemented as a mechanism for cross-jurisdictional drug access, and what institutional outcomes has it achieved? What persistent structural challenges limit its effectiveness and long-term sustainability? And how should the program evolve in governance architecture, real-world evidence (RWE) infrastructure, and financing design to realize its potential as a model of adaptive regulatory governance? By applying a delegated regulatory governance lens, this English-language synthesis fills a critical gap in the international literature (20, 21).

## 2 Scope and evidence base

This review followed the PRISMA Extension for Scoping Reviews (PRISMA-ScR) guidelines. We searched three English databases (PubMed, Embase, ScienceDirect) and three Chinese databases (CNKI, Wanfang Data, VIP) for records published from database inception to 30 May 2026. The initial search was conducted on 2 March 2026 and updated on 30 May 2026 immediately before submission. Gray literature was identified through manual searches of the official websites of relevant national and provincial authorities, including the NMPA, the National Health Commission, and the governments and drug administrations of Guangdong and Hainan provinces.

Eligible records were peer-reviewed studies, governmental policy documents, and industry reports addressing the design, implementation, regulatory framework, or clinical application of the HKMEC policy and related GBA cross-jurisdictional drug-access mechanisms, including work on real-world data governance, drug selection, and comparisons with other subnational pilots such as Lecheng; conference abstracts, non-primary commentaries, duplicates, studies without an HKMEC/GBA regulatory focus, non-Chinese/English literature, and records without retrievable full text were excluded. Of 163 records initially identified, 53 records and official documents were included after deduplication and stepwise screening.

The included corpus spans heterogeneous source types—peer-reviewed empirical studies, official government instruments, institutional reports, and gray literature—rather than a homogeneous body of intervention studies. Consequently, formal instruments such as GRADE, which are designed to rate the certainty of effect estimates from clinical studies, were not directly applicable. Consistent with PRISMA-ScR, which does not mandate formal risk-of-bias assessment for scoping/policy reviews, we instead applied a transparent source-tiered appraisal scheme adapted to policy-review evidence. Each included source was classified into one of four evidentiary tiers (T1, peer-reviewed empirical or real-world-evidence studies; T2, official government instruments and gazetted catalogs; T3, authoritative institutional reports, expert consensus statements, and policy analyses; T4, conference proceedings, theses, and industry or news items), each with a defined permitted evidentiary use (Supplementary Table S1). T1–T2 sources could support summative or quantitative claims; T3 could support contextual and interpretive statements; and T4 was restricted to narrative background and was not used to underpin claims of policy effectiveness. Tier assignment was performed by one reviewer and verified by a second, with disagreements resolved by discussion; the tier of every included source is reported in Supplementary Table S2.

## 3 Policy framework and governance structure

### 3.1 Regulatory architecture and policy design

From a comparative perspective, the closest international analogs are Australia’s Provisional Approval pathway (22) and the EU’s Conditional Marketing Authorization, both of which grant provisional market access ahead of a complete evidence package, subject to post-authorization evidence obligations (23, 24). A stronger functional parallel may be Japan’s Sakigake designation (18), which relies partly on evidence generated in foreign regulatory processes to expedite domestic review for priority drugs (25). HKMEC departs from these comparators in one key respect, however: approval does not entail an independent scientific reassessment by the NMPA; the Hong Kong or Macao listing decision instead serves as the evidentiary gateway, with Guangdong’s role limited to administrative approval. This design achieves access speed at the cost of direct NMPA scientific evaluation. The trade-off is defensible because the Chinese government formally accepts Hong Kong and Macao regulatory standards as adequate proxies.

Three successive instruments have governed the program. The GBA Drug and Medical Device Regulatory Innovation Development Work Plan established the framework, scope, and phased implementation logic, targeting basic institutional mechanisms by 2022 and full regulatory coordination by 2035 (9). The Interim Regulations on Administration of Clinically Needed Imported HK/Macao Drugs and Medical Devices in GBA Mainland Areas (August 2021; Guangdong PAMR [2021] No. 4) provided the operational rulebook: conditions for institutional designation, a two-tier approval scheme distinguishing catalog from off-catalog products, risk management obligations, adverse-event reporting chains, and end-to-end traceability requirements (26). The December 2024 Guangdong Provincial Regulations superseded the Interim Regulations, conferring statutory (rather than merely normative) force on the entire program and, critically, opening the pathway for HKMEC-generated RWE to support national drug registration (Article 23) (27).

The HKMEC governance structure spans three administrative tiers (9). At the national level, the NMPA retains overall regulatory framework authority, including the issuance of RWE methodological standards applicable to regulatory decision-making and external data integration; its Drug Evaluation Center has issued RWE standards applicable to HKMEC-generated data (28), and the GBA sub-center provides regional technical liaison. At the provincial level, the Guangdong Provincial Drug Review Board (GDPDRB) manages the product catalog, convenes expert evaluation panels, and publishes approved batches, while the Guangdong Provincial Health Commission designates hospitals. Municipal commissions handle day-to-day surveillance and adverse-event follow-up. Between the regulatory hierarchy and clinical sites sits the licensed importer-distributor, run exclusively by China Resources Guangdong Pharmaceutical Co., Ltd. during the pilot phase (12). Designated medical institutions bear the highest accountability: the policy explicitly assigns them “full responsibility for the safety risks of imported products” (3, 15), requiring dedicated HKMEC pharmacies with independent storage and temperature monitoring, prescribing restrictions to associate-chief-physician level or above with documented product-specific training, and periodic pharmacovigilance reviews reportable to both provincial and municipal regulators. The comprehensive governance model at HKU-SZH [encompassing drug selection criteria (15), RWD quality management (14), zero-markup pricing, ethics committee oversight, and bilingual informed-consent documentation (29)] remains the program’s most elaborated institutional template. Replicating it across 71 sites of varying capacity, however, remains an ongoing challenge.

### 3.2 Chronological policy milestones

Table 1 summarizes the major regulatory and institutional events from the program’s inception through early 2026. The trajectory shows a deliberate “test-and-expand” strategy, with each expansion phase contingent on demonstrated safety and operational feasibility at existing sites. While this approach aligns with principles of incremental regulatory governance, it has also led to an uneven distribution of capacity across the network.

**Table 1:** Major milestones in HKMEC policy development (2020–2026).

Period	Event	Substantive content
Sep 2020	Work Plan enacted	Eight ministries jointly issued the Work Plan for Regulatory Innovation and Development of Drugs and Medical Devices in the GBA. The State Council authorized the Guangdong Provincial Government to exercise approval authority (ordinarily vested in the NMPA) for clinically needed HK/Macao-listed drugs at nine inland GBA cities.
Jan 2021	Pilot activated at HKU-SZH	Nine Guangdong departments jointly relayed the Work Plan. HKU-SZH designated as the sole pilot site, with the trial period running through 31 July 2021.
Aug 2021	Expansion phase; Interim Regulations promulgated	Interim Regulations formalized designation conditions, approval procedures, risk-management obligations, and end-to-end traceability requirements. Five first-batch institutions announced; China Resources Guangdong Pharmaceutical appointed sole licensed importer-distributor.
Jun 2022	Mainland manufacturing pathway for HK/Macao MAHs established	NMPA issued implementation plans supporting HK/Macao Marketing Authorization Holders (MAHs) to manufacture drugs and medical devices at GBA mainland facilities, extending the HKMEC ecosystem into the production domain.
Feb 2023	Second-batch institutions (14 hospitals)	Coverage extended to six of nine GBA inland cities. Catalogs 1–4 had by this date approved 20 drugs and 12 devices. Product engagement among newly designated institutions lagged significantly behind HKU-SZH.
Sep 2024	Third-batch institutions (25 hospitals); 7th catalog batch	All nine GBA inland cities now covered (45 designated sites). Seventh catalog batch added 10 drugs and 10 devices, bringing cumulative approved products to 92.
Dec 2024	Provincial Regulations enacted (effective 1 Dec 2024); 2024 Edition Catalog released	Guangdong Provincial People’s Congress enacted the Regulations on Administration of Imported HK/Macao Drugs and Medical Devices in the Nine GBA Inland Cities, upgrading HKMEC from administrative pilot to statutory law and establishing a permanent three-jurisdiction regulatory coordination mechanism. The 2024 Edition Catalog (71 items: 34 drugs and 37 devices) was concurrently released.
Dec 2025	Fourth-batch institutions (27 hospitals)	With the addition of the fourth batch, 71 designated medical institutions are now approved under the HKMEC program. By this point, 140 products (63 drugs and 77 devices, cumulative total since inception) have been authorized for import and clinical use.
Feb 2026	2025 Edition Catalog released	The 2025 Edition Catalog comprises 115 products (45 drugs and 70 medical devices), representing an increase of 11 drugs and 33 devices compared with the 2024 Edition.

HKU-SZH, The University of Hong Kong-Shenzhen Hospital; PAMR, Provincial Administration for Market Regulation; Data are current as of May 2026, compiled from official releases by NMPA and GDMPA (51). GDMPA, “Special Column on Regulatory Innovation and Development of Drugs and Medical Devices in the Guangdong–Hong Kong–Macao Greater Bay Area”. Available at: http://mpa.gd.gov.cn/ztzl/dwq/ (accessed May 2026).

## 4 Implementation record: products, institutions, and clinical outcomes

### 4.1 Approved drug and device catalogs

Across seven successive product batches and two consolidated edition catalogs, the HKMEC range has expanded steadily. The current 2025 Edition Catalog—published on 2 February 2026 and superseding the 2024 edition (30)—supersedes the 71 cataloged items (34 drugs and 37 devices) listed at the end of 2024 (31). As of the 2025 Edition, the in-use catalog comprised 115 products (45 drugs and 70 devices)—i.e., products currently active in the catalog—while the cumulative number of products approved since the policy’s inception in 2020 reached 140 (63 drugs and 77 devices) (13); the difference reflects products that have exited the in-use catalog (e.g., through transition to standard registration or non-renewal at pilot expiry). The 2024 edition comprised 71 cataloged products in total. Per the issuing notice, these were drawn from two sources within a single unified catalog: Hong Kong/Macao-listed products published in accordance with the 2018 Announcement No. 79 on the review and approval of clinically urgent overseas new drugs and meeting the Regulations’ requirements, together with products that had already been approved before the Regulations took effect. All listed products have been administered under Article 9 of the Regulations since 1 December 2024. The “clinically urgent overseas new drugs” designation therefore refers to this national review-and-approval framework for qualifying Hong Kong/Macao-listed products, not to a separate category or exception to the listing criterion (32).

The drug portfolio skews toward oncology, which accounts for roughly 42% of approved pharmaceuticals (33). On the device side, the magnetically controlled growing rod (MCGR)—the first device approved and used under HKMEC, implanted in May 2021 at HKU-SZH in a nine-year-old with early-onset scoliosis (12)—enables non-invasive distraction via an external remote controller, replacing the repeated open surgical lengthenings required by conventional growing rods and reducing the treatment course by approximately 10 procedures.

### 4.2 Designated institutions: network expansion and participation disparities

**Figure 1:**
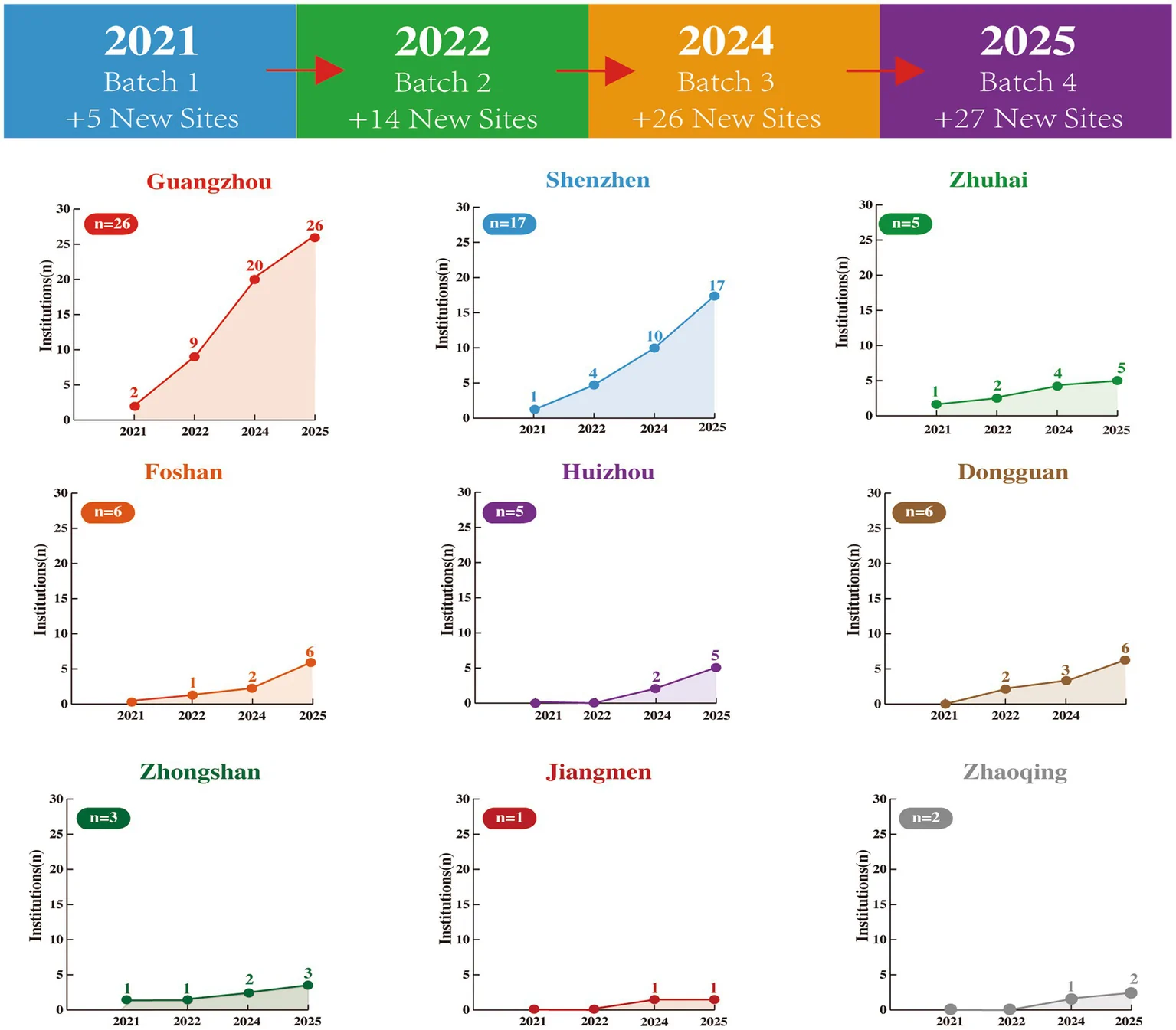
Cumulative designated institutions by city and policy batch. The x-axis reflects the years in which successive batches of HKMEC designated medical institutions were announced. The Guangdong Provincial Health Commission and the Guangdong Provincial Medical Products Administration released the first to fourth batches of the designated-institution list in 2021, 2022, 2024 and 2025, respectively; no new batch of designated institutions was announced in 2023, which is why no data point appears for that year.

**Figure 2:**
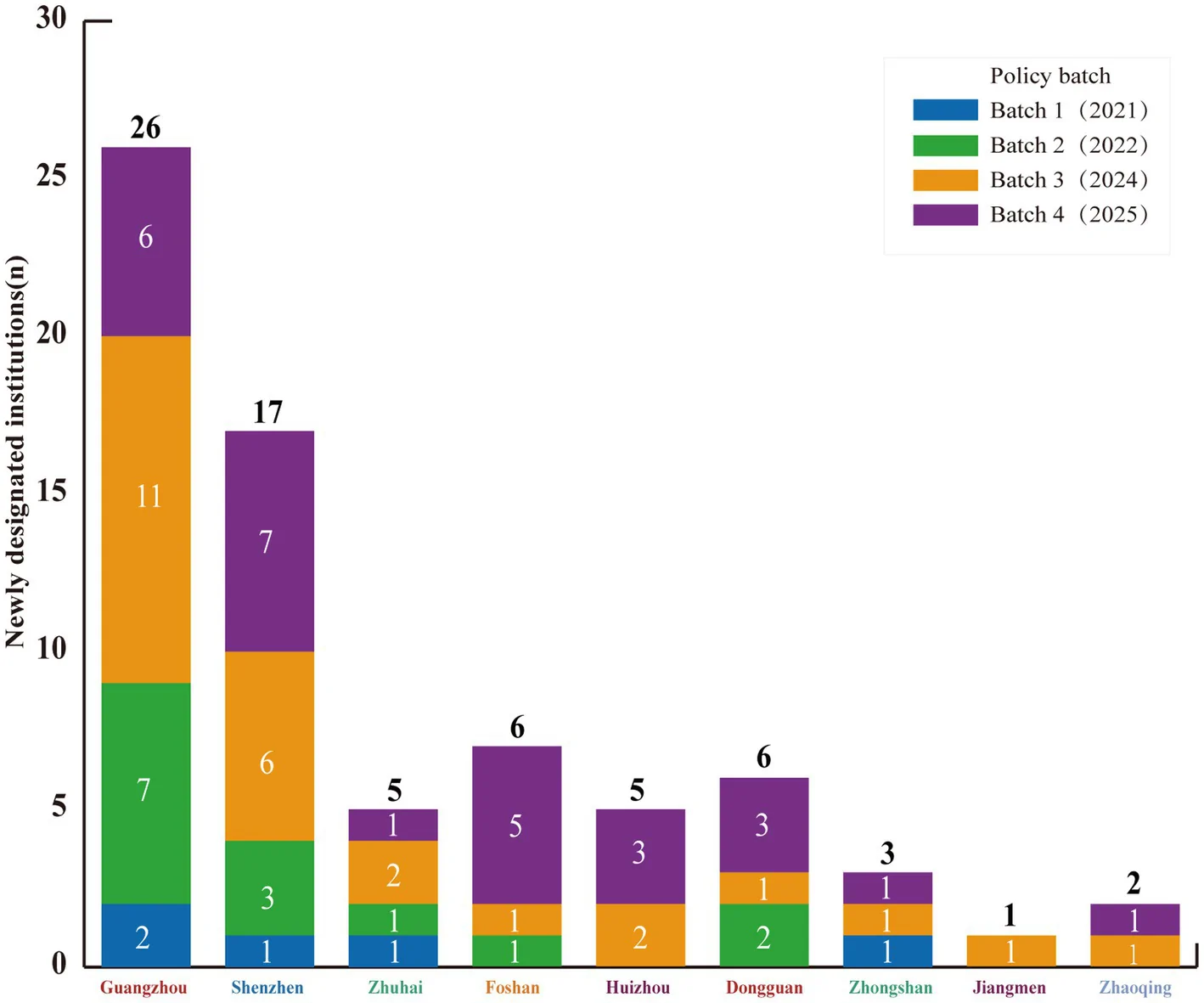
Cumulative number of designated institutions by city. In Batch 3 (2024), due to qualification changes, Zhuhai has 2 designated medical institutions: The Fifth Affiliated Hospital of Sun Yat-sen University and The First Affiliated Hospital of Guangzhou Medical University, Hengqin Hospital. In Batch 4 (2025), one Foshan designated institution was de-designated, reducing cumulative active Foshan total to 6 institutions. The total number of active HKMEC designated medical institutions across the nine GBA cities is therefore 71 as of 2025. Source: Guangdong Provincial Health Commission.

Designation of hospitals has proceeded in four tranches. The pioneer group (August 2021) comprised five institutions in Shenzhen and Guangzhou, with HKU-SZH occupying a category of its own, owing to its joint University of Hong Kong and mainland governance structure and prior experience importing drugs for its cross-border patient population. The second batch (February 2023) added 14 hospitals from six cities; the third (September 2024) expanded by 25 further sites, achieving full coverage of all nine GBA inland cities; and the fourth batch (December 2025) added 27 more, bringing the cumulative total to 71 designated hospitals. Figure 1 shows the cumulative growth trajectory of designated institutions in each city across successive policy batch. Guangzhou hosts the densest concentration (26 institutions), followed by Shenzhen (17), while the remaining seven cities collectively host 28 sites, several with only one or two. Figure 2 presents the same cumulative totals decomposed by the policy batch in which each institution was designated.

Geographic coverage, however, masks stark asymmetries in actual participation. HKU-SZH has introduced 16 drugs and 11 devices as first applicant, substantially more than any other site, whereas multiple second-batch hospitals had introduced no products at all at the time of Sun et al.’s (31) analysis in late 2024. Four factors explain this divergence. First, HKU-SZH benefited from the institutional advantages of pioneer status, early policy endorsement, and streamlined regulatory procedures. Second, only well-resourced hospitals can sustain compliant HKMEC pharmacies. Third, familiarity with Hong Kong formularies is rare outside cohorts with cross-border clinical experience. Finally, hospital governance that prioritizes innovation creates strong internal motivation.

### 4.3 Patient access and clinical outcomes

Aggregate patient-access figures have grown substantially year on year. By August 2024, the first two batches of designated institutions (19 sites) had collectively introduced 80 product types and recorded more than 8,000 patient encounters; relative to August 2023, this represented over 85% growth in product variety and more than 200% growth in utilization (34). Total encounters across the full network exceeded 17,000 by early 2026. Approximately 70% of current users are mainland GBA residents rather than Hong Kong or Macao patients, a demographic distribution that reflects both the preponderance of mainland-affiliated institutions in the network and the availability of publicly subsidized alternatives for HK/Macao residents within their home jurisdictions. As an illustrative early-use example, a single-center case series at Zhongshan Chenxinghai Hospital reported reductions in monthly headache days in a majority of migraine patients treated with erenumab accessed through HKMEC (34). This observation is exploratory: it derives from a single center, lacks a comparator arm, and the source does not disclose the sample size. It should therefore be read as preliminary single-center experience requiring rigorous, controlled validation, rather than as evidence of the policy’s clinical effectiveness.

## 5 Research advances: a domain-by-domain synthesis

### 5.1 Policy implementation: institutional mechanics and distributional effects

Huo et al. (12) provided one of the field’s first systematic implementation studies, applying the Meter-Horn Policy Implementation System Model to trace China Resources Guangdong Pharmaceutical’s operational evolution across three sequential phases. The first phase revealed that establishing the green-channel customs clearance mechanism required months of joint coordination among four departments: Guangzhou Customs, Shenzhen Customs, the Guangzhou Port Drug Supervision Office, and the Guangdong Provincial Drug Administration. Only after this effort was a feasible operating procedure finally formulated. It also reflects that there is no mature unified administrative standard for the customs clearance of medicines that have not obtained domestic registration approval and official import permits issued by NMPA. The second phase documents the construction of a proprietary end-to-end traceability system (“one item, one code”), developed precisely because national drug traceability standards apply only to registered drugs; this platform was purpose-built by a commercial distributor filling a regulatory gap that government agencies had not yet closed. The third phase traces the transition from Hong Kong-only procurement to direct global sourcing, requiring headquarters-level engagement with Roche, Pfizer, AstraZeneca, and other manufacturers.

Lin and Gao (34) extended the implementation analysis to structural and economic dimensions. Even for drugs already on the approved HKMEC catalog, each newly designated hospital must complete a full *de novo* institutional application: submitting product dossiers, convening internal medical committee evaluations, presenting expert justifications to provincial reviewers, and obtaining fresh regulatory approvals, with no cross-institutional recognition of prior approvals. This process typically consumes over a year from application initiation to first patient use at pioneer sites. The paper also documents an underappreciated dynamic. Mainland physicians know little about Hong Kong formulary practice. Visiting HK physician consultations are limited to one or two sessions a month by cost and logistics. As a result, a small group of cross-border-experienced practitioners decides which drugs get introduced—and the bottleneck becomes systemic.

### 5.2 Real-world data: quality standards, platform architecture, and the regulatory conversion gap

The HKMEC program generates a unique dataset: the first clinical use of specific drugs in Chinese patient populations outside the artificial constraints of a registration trial. A key unresolved scientific question is whether this dataset can be converted into regulatory-grade RWE. Such evidence would need to be suitable for supporting future NMPA registration submissions (35).

Zhou et al.’s (14) prospective quality audit at HKU-SZH remains the only published systematic empirical assessment of HKMEC RWD quality to date. Analyzing 99 patient records collected between April 2021 and November 2022, they found in multivariate analysis that assigning dedicated per-product data monitors substantially reduced the incidence of data quality problems. This suggests that the primary remedy lies in human resource investment in data quality oversight rather than in technological solutions alone. This lesson has direct implications for scale-up across the broader network.

At the system level, Sun et al. (31) identified several structural impediments to HKMEC RWD serving as a meaningful regulatory pipeline. The first is data fragmentation: each institution’s clinical data system operates independently, with non-standardized data dictionaries and no interoperability, precluding cross-center pooling. The second is a governance-purpose misalignment (36): existing HKMEC studies are predominantly designed as physician-initiated academic investigations with peer-reviewed publication as the primary output, rather than regulatory-grade studies designed from inception to meet NMPA evidentiary standards. The third is resource concentration: HKU-SZH generates the substantial majority of all documented HKMEC RWD, creating both data representativeness and adverse-event detection limitations. By early 2026, no HKMEC product has yet been documented as supporting an NMPA registration submission with GBA-generated RWE. This contrasts sharply with Lecheng, where 17 products had supported NMPA submissions and 40 more were in active RWD studies as of November 2024 (31). The gap persists despite Article 23 authorization in the 2024 Provincial Regulations. The reason is that most routine clinical records fall short of regulatory-grade standards.

### 5.3 Drug selection: constructing evidence standards for unregistered products

Li et al. (15) at HKU-SZH developed the field’s most operationally detailed response to the drug selection challenge: a six-dimension, 15-indicator selection model explicitly calibrated to the unregistered-product context. Three core characteristics deserve close attention. Firstly, the urgency criterion fully takes clinical practical demand into account. It covers unmet medical needs, lack of alternative domestically approved medicines and epidemiological application value, which differs greatly from conventional health technology evaluation systems. Such mainstream frameworks mainly focus on therapeutic effect comparison instead of formulating targeted market access policies (16). Secondly, its safety evaluation part emphasizes thorough analysis of drug safety data sourced from Asian groups. This approach can better reflect real safety conditions among Chinese patients, instead of simply adopting overall research results obtained mostly from Western trial subjects. Thirdly, the social value evaluation focuses on judging whether medicines introduced via HKMEC can make up for domestic registration shortages that will remain unsolved in the next 2 to 3 years. This practical time-based screening rule prevents approving drugs that will soon enter the mainland through official regular channels before they are widely used under the HKMEC scheme.

### 5.4 Clinical comprehensive evaluation: the MCDA framework and its adaptations

Cailin et al. (16) conducted a systematic review of six major drug value assessment frameworks (NICE-VAF, ICER-VAF, IQWiG General Methods, China’s Drug Clinical Comprehensive Evaluation (DCCE) guideline, PPVF, and MCDA) and concluded that no existing international framework can be applied to HKMEC products without substantial modification. The NICE and ICER frameworks assume an established national-population evidence base, an assumption violated by every HKMEC product. IQWiG additionally requires comparative assessment against an “appropriate comparator therapy” that simply does not exist for rare-disease indications with no licensed mainland alternative. China’s own DCCE guideline lacks a defined evaluation pathway for unregistered products entirely.

MCDA emerges as the most adaptable framework: its dimensional structure can be configured rather than applied as-is; its output is a quantitative composite score amenable to cross-product comparison; and its multi-stakeholder weight-assignment methodology can explicitly incorporate HKMEC-specific dimensions, including clinical urgency as a first-order selection criterion, cross-border access convenience as a policy-attribute dimension, and individual patient financial burden as a separate economic sub-indicator not captured in standard ICER or NICE economic modeling. Cailin et al.’s (16) framework remains to be validated through formal consensus methods such as Delphi, which constitutes an important near-term research priority given the program’s expanding catalog and growing pressure on designated institutions to justify resource allocation decisions.

### 5.5 Legislative scholarship and the theory of provincial advance legislation

Liu and Zhu (37) situate the HKMEC policy within the specific constitutional and administrative law niche of provincial advance legislation. This category is authorized by Article 93 (5) of China’s Legislation Law when formal provincial regulations are not yet mature but administrative urgency is demonstrable. Their argument that the program satisfied this threshold rested on three claims: the demonstrable and unmet medical need that the program addresses; the formal gap in national law (neither the Drug Administration Law nor the Medical Device Supervision and Administration Regulations explicitly authorizes or prohibits the delegated-authority model); and the accumulated safety track record providing the empirical basis for converting pilot experience into statutory permanence. The December 2024 Regulations fulfilled this anticipated trajectory.

## 6 Comparative analysis: HKMEC and the Hainan Lecheng pilot zone

China’s two sub-national pharmaceutical access programs—HKMEC in the GBA and the special drug policy at the Boao Lecheng International Medical Tourism Pilot Zone (Hainan Province)—share the same originating logic (State Council-delegated approval authority for clinically urgent unregistered products), the same core mechanism (designated-institution use without NMPA registration), and partially overlapping product portfolios. Yet they operate in fundamentally different institutional niches, and understanding these differences is essential for developing a coherent national strategy for expedited access. Table 2 provides a structured comparison across nine parameters.

**Table 2:** Comparative analysis of the HKMEC program and the Hainan Lecheng international medical tourism pilot zone.

Parameter	HKMEC program (GBA)	Lecheng pilot zone (Hainan)
Regulatory delegation (9, 52)	Guangdong Provincial Government (authorized by State Council under the Regulations on Formulation of Administrative Regulations)	Hainan Provincial Government (authorized by State Council decisions of 2018 and 2019)
Product eligibility (26, 38)	Must already be listed and actively used in HK or Macao public hospitals; clinically urgent; no adequate mainland registered alternative	Approved in any ICH-member jurisdiction; not yet registered in China; no domestic marketed equivalent; excludes vaccines and certain biologics
Approval speed (33)	Post-2024 Regulations: 10 working days (catalog items); 20 working days (off-catalog). In practice, median time from institutional application to first patient use has exceeded 1 year at pioneer sites	First-time application: typically days to weeks. Non-first-time: mean 1–3 working days; fastest approvals within hours. Batch-approval model for catalog products
Geographic scope (33)	71 hospitals across all nine GBA inland cities; urban agglomeration population >70 million; patient volumes vastly exceed Lecheng	Geographically concentrated within Lecheng zone, Qionghai, Hainan; dedicated medical-tourism ecosystem with a substantially smaller patient pool
RWD/RWE pathway (53)	Encouraged by 2024 Regulations (Article 23); no unified cross-institutional data platform; institutional data remain siloed. By early 2026, no HKMEC products have supported an NMPA registration submission with zone-generated RWE to date	National RWD pilot since 2022; formal three-party mechanism (NMPA CDE + Hainan PDRA + Lecheng Bureau); centralized NMPA-standard platform. As of November 2024, 17 products used Lecheng RWE in NMPA review; 40 products in active RWD studies
Insurance coverage (33)	All HKMEC drugs self-pay; Guangzhou and Zhuhai operate inclusive commercial health insurance pilots covering select products; 2024 Regulations encourage but do not mandate replication	“Lecheng Global Special Drug Insurance” in operation: no age or occupation limits, no waiting period, no deductible, no pre-enrolment medical check; substantially lowers individual financial burden
Customs model (12)	Standard port customs; typical clearance 2–3 weeks per shipment; each consignment requires individual clearance documentation	“Pre-entry, batch-inventory; per-use, batch-clearance” model; drugs are cleared en masse into the bonded warehouse; clearance is often same-day
Legislative basis (27)	Provincial Regulations (effective Dec 2024): the first statutory law in China specifically governing cross-border SAR drug importation; confers permanent legal basis replacing time-limited Interim Regulations	State Council decisions (2018/2019); Hainan provincial regulations (2023); Hainan Free Trade Port special-zone law: a multi-layered, well-established legal architecture
Core competitive advantage	Population scale and geographic breadth; proximity to HK/Macao clinical practice; capacity to generate one of the largest Chinese-patient RWD datasets on novel international drugs (contingent on maturing data infrastructure)	Approval speed; broadest product eligibility of any Chinese zone; most mature RWE-to-registration pipeline; well-developed insurance product; highly streamlined customs mechanism

ICH, International Council for Harmonization; NMPA CDE, National Medical Products Administration Center for Drug Evaluation; PDRA, Provincial Drug Regulatory Administration; SAR, Special Administrative Region. Data are current as of May 2026 based on official releases from NMPA and Guangdong Provincial Drug Regulatory Bureau.

Approval speed is a major operational difference. Lecheng’s non-first-time approval typically completed within 1–3 working days, fastest within hours. Its efficiency derives from a zonal administration model that integrates medical oversight and pharmaceutical regulation within a single institutional authority, enabling parallel processing that HKMEC’s multi-tiered provincial structure may have difficulty replicating. HKMEC’s mandatory 10-working-day timeline for catalog products, while a substantial improvement over previous practice, still reflects the sequential review requirements of a geographically dispersed system with separate provincial- and municipal-level actors.

HKMEC’s distinctive structural strength lies in patient scale and geographic reach. The 71 designated hospitals in a continuous urban agglomeration of more than 70 million people generate patient volumes, and therefore potential RWD datasets, on a scale that Lecheng’s geographically isolated, medically specialized tourist medicine model cannot approach. The implication is that HKMEC, if it developed data governance infrastructure equivalent to Lecheng’s, could potentially generate one of the largest bodies of Chinese-patient clinical use data on novel international drugs. The Lecheng experience proves this is achievable: its 17 RWE-supported NMPA submissions and 40 active RWD studies as of November 2024 reflect disciplined governance design rather than patient volume alone (38). Sun et al. (31) proposed formal mutual product evaluation recognition between the two programs as the most immediate practical opportunity, while Li et al. (3) recommended adopting Lecheng’s batch-clearance customs model across GBA ports.

## 7 Persistent challenges and their structural roots

### 7.1 The self-pay model: financial barriers and market dynamics

All HKMEC drugs are, by program design, currently self-funded by patients (though limited commercial insurance coverage has been piloted in Guangzhou and Zhuhai). Prices are set by reference to HK/Macao public hospital procurement prices. This is a legitimate choice that prevents markup by designated institutions (39) but it does not address the fact that novel biologics and targeted therapies are expensive at any reference price. Most HKMEC products carry treatment costs from several thousand to hundreds of thousands of yuan per course, placing them well beyond the financial reach of the majority of GBA residents who lack supplementary commercial insurance. The program’s own utilization data confirm this: approximately 70% of current users are mainland GBA residents with sufficient economic resources to self-fund treatment, rather than the most clinically underserved populations that the access-equity rationale of the program implies.

Market dynamics create an additional financial risk. Several HKMEC drugs have subsequently been included in China’s national centralized volume-based procurement (VBP) program, which has been shown to substantially reduce drug prices through “volume-for-price” mechanisms and large-scale purchasing power (40, 41). As a result, the prices of domestically registered alternatives have declined dramatically following inclusion in procurement rounds, reshaping market competition and eroding the price advantage of cross-border accessed products (40, 42). Hospitals holding HKMEC inventory face stranded-cost exposure; physicians become reluctant to initiate HKMEC therapy when procurement alternatives are imminent. These dynamics underscore the need for rigorous product portfolio discipline that prioritizes products with a sustained registration gap.

### 7.2 Product portfolio discipline: clinical urgency as a selection standard

Sun et al.’s (31) product-level analysis identified a subset of approved HKMEC drugs whose claimed clinical urgency warrants scrutiny. Several approved products had received their global first approval more than a decade before HKMEC introduction, raising questions about whether the clinical need they address is more accurately characterized as longstanding domestic regulatory neglect than acute medical emergency. More operationally troubling is a recurrent pattern: products received HKMEC approval, were adopted by only one or two institutions, and then received NMPA national approval within months. This sequence generated only a minimal body of patient exposures and contributed neither meaningful RWD nor clinical insight beyond what standard registration would have produced. For these products, HKMEC essentially failed to deliver on its pilot function—they were never tested at scale in real-world conditions before national approval arrived.

The 2025 Application Guidelines require explicit clinical urgency justification: applicants must specify the absence of domestic registered equivalents, quantify the patient population without alternative access, and project the expected NMPA registration timeline. This requirement provides a procedural mechanism for filtering such applications at entry. Two further requirements would sharpen portfolio discipline. First, applicants should demonstrate awareness of any pending NMPA application for the same product. Second, they should provide a credible estimate of the minimum HKMEC utilization period before national approval is granted.

### 7.3 Real-world data ecosystem: the infrastructure gap

A central tension in HKMEC’s RWD system lies between the scale of data generated by the program and the governance infrastructure required to translate it into regulatory-grade evidence. HKU-SZH launched China’s first specialized RWD management platform for HKMEC initiatives. It has gathered clinical records of numerous drugs from thousands of patient visits. Even so, the platform operates independently within a single hospital. Relevant data cannot be merged into regional shared databases, nor shared among designated hospitals, and cannot fully satisfy the data standard specifications for NMPA (14, 28). The 2024 Regulations’ Article 19 mandate for a unified monitoring platform and Article 23’s RWE-to-registration provision create the statutory basis for closing this gap (27); what is required now is a concrete implementation program with defined timelines, technical standards, and resourcing.

### 7.4 The depth problem: medical integration vs. drug importation

Scant attention has been paid to a structural limitation in HKMEC’s design: the gap between the program’s integration ambitions and operational reality. While the policy envisions deeper clinical linkages with Hong Kong and Macao, most designated mainland hospitals in practice maintain only limited working ties with HK/Macao clinical systems. Drug importation and medical integration are conceptually distinct. The current model relies heavily on visiting HK physician consultations, which are constrained to one to two sessions per month by transportation costs and logistical complexity, to enable the clinical knowledge transfer required for mainland physicians to prescribe HKMEC drugs appropriately. Cross-border electronic health record interoperability remains limited at scale, with only isolated pilot implementations such as Shenzhen’s blockchain-based cross-border EHR pilot at HKU-SZH (13). The systemic incompatibility between Hong Kong’s “general practice first, then specialist” referral model (43, 44) and mainland China’s specialist-first access system (34, 45) creates additional structural barriers for HK/Macao residents seeking care at HKMEC institutions. This barrier limits both utilization by the target population and the depth of clinical learning that the program’s unique access position could otherwise generate (13).

### 7.5 Pharmacovigilance and safety surveillance

A structured institution-level pharmacovigilance framework already exists under HKMEC: at HKU-SZH, a real-world-data quality-management system records adverse events against six core elements (event name, onset, resolution/outcome, severity, causality, and concomitant medications), standardized to international vocabularies (ICD-10, SNOMED-CT, MedDRA, LOINC) (14), with cross-boundary exchange operating through the traceability platform established under the 2024 Regulations (27). No comprehensive HKMEC adverse-event dataset has been published over the five-year period, however, and neither the events detected across the network nor the multi-party data-exchange protocols (what is shared, by whom, on what schedule, and under what governance) are in the public record. We flag this as a critical evidence gap rather than an indication of safety, and recommend a transparent, periodically published cross-border pharmacovigilance mechanism with a formalized data-exchange protocol. A feasible technical pathway is active, real-world-data-based surveillance: standardizing front-line clinical data to a common data model—such as the OMOP CDM, with mappings to RxNorm, SNOMED-CT and LOINC and derived structures (e.g., the Drug Era table) for precise exposure specification (46)—would enable reproducible signal detection combining descriptive (US-Sentinel-type) analyses, disproportionality methods, and sequential monitoring, building on the vocabulary standardization already piloted under HKMEC (14). Within such a framework, clinical pharmacists can be assigned defined functions (prescription review, medication reconciliation, structured adverse-reaction reporting, and patient education), conditional on the prior data-quality governance and cross-border interoperability that remain to be established.

Institution-level pharmaceutical-care practice across the network can be characterized only partially, and the available sources support a differentiated comparison rather than a uniform assumption. At the network level, adverse-event reporting is governed by a pair of provincial work guidelines issued under a single 2025 notice of the Guangdong Center for Adverse Drug Reaction Monitoring—covering imported Hong Kong and Macao drugs and medical devices, respectively, in the nine GBA mainland cities—which oblige every designated institution to maintain a monitoring body, a responsible person, and an ADR analysis team, conduct staff training, and report to the prefecture-level agency (47). Against this common baseline, documented practice diverges in maturity: the most operationally detailed public account (Guangzhou First People’s Hospital, Nansha campus) reports a three-committee “HKMEC working group,” a closed-loop procurement-to-disposal workflow modeled on narcotic-drug controls, pharmacist-led follow-up and recall, and dual ADR/adverse-event monitoring via a dedicated HKMEC module in the hospital safety-event system (48)—closely paralleling the HKU-SZH template and indicating that the flagship model is at least partially transferable to well-resourced tertiary institutions. For most other second- and third-batch institutions, comparable detail is not publicly available; where it is not, we state this explicitly rather than extrapolating from HKU-SZH. Two conclusions follow. First, the dedicated per-product data-monitor and dedicated-module arrangement—shown at HKU-SZH to reduce data-quality problems (14) and mirrored at Nansha—is a replicable organizational design rather than an institution-specific idiosyncrasy. Second, the principal structural bottleneck is the absence of standardized minimum pharmaceutical-care requirements: these provincial guidelines standardize safety reporting but do not prescribe a minimum clinical-pharmacy service specification, leaving prescription review, reconciliation, and patient education to institutional discretion. Establishing and publicly reporting such baseline standards across all designated institutions would both close this evidence gap and reduce inter-institutional heterogeneity.

## 8 Legislative consolidation: what the December 2024 regulations change

The December 2024 Regulations (27) represent not an incremental improvement but a qualitative change in the program’s legal status and governance architecture. The five most consequential substantive provisions are: (1) Article 5 (permanent three-jurisdiction coordination mechanism), which elevates Guangdong-HK-Macao regulatory collaboration from *ad hoc* to institutional; (2) Article 9 (statutory approval timelines of 10 and 20 working days), which converts targets into legally binding obligations subject to administrative accountability; (3) Article 19 (mandatory monitoring platform and unique product identification), which creates the technical infrastructure prerequisite for cross-institutional data interoperability; (4) Articles 7–8 (codified expert database and periodic/dynamic catalog adjustment), which provide due-process legitimacy to the selection mechanism; (5) Article 23 (explicit RWE-to-registration provision), which, if operationalized with specific NMPA technical standards, could fundamentally transform the program’s relationship with the national regulatory evidence base. The Lecheng experience (17 registration-supporting RWE submissions generated through its structured three-party mechanism as of November 2024) illustrates the magnitude of what Article 23 could unlock if similarly institutionalized.

## 9 Actionable recommendations

Five years of implementation confirm the program’s achievements (meaningful lead-time advantages, an expanding product network, and more than 17,000 documented patient encounters) alongside structural limitations that require deliberate reform: fragmented real-world data infrastructure, exclusively self-pay financing, uneven institutional capacity, and shallow cross-border medical integration. Five priority recommendations address these in turn. Indicative implementation specifications for each recommendation—including proposed lead entities, collaborating parties, phased KPIs, and timelines—are summarized in Table 3.

**Table 3:** Indicative implementation specifications (RACI-style) for recommendations 9.1–9.5.

Element	Content (authors’ proposal)
9.1 Build a regulation-grade RWD infrastructure	
Lead (R/A)	Guangdong Provincial Medical Products Administration (GDMPA) and Guangdong Provincial Health Commission (GDHC), jointly (proposed lead)
Collaborating (C)	The 71 designated institutions; NMPA Center for Drug Evaluation (CDE); RWD technical partners
Informed (I)	NMPA; GBA municipal authorities
Governance model	Formal three-party cooperation mechanism, modeled on Lecheng (NMPA CDE - Hainan PDRA - Lecheng Bureau), with legally specified roles, data-access protocols, and NMPA submission procedures
Phase 1 (0–24 mo) KPI	Regulation-grade RWD platform built to NMPA standards with OMOP-CDM compatibility; standardized data integration across > = 50% of designated institutions
Phase 2 (24–36 mo) KPI	Submission of the first GBA-data-based NMPA registration-support dossier
Timeline	~24-month construction horizon (benchmark: Lecheng platform initiated 2020, operational 2022); 36-month dossier milestone
9.2 Streamline processes and develop institutional capacity	
Lead (R/A)	GDMPA (process reform); GDMPA-coordinated capability program (proposed lead)
Collaborating (C)	GBA customs (port clearance); pioneer hospitals (peer-support network); the 27 fourth-batch institutions
Informed (I)	GDHC; designated-institutions network
Phase 1 KPI	‘Pre-entry, batch-inventory; per-use, batch-clearance’ customs model adopted across GBA ports; per-shipment clearance reduced from weeks to days
Phase 2 KPI	Notification-and-verification (self-certify + post-initiation audit) model operational for catalog-listed products; standardized pharmacy-setup checklists, prescriber-training modules and informed-consent templates delivered to all 27 fourth-batch institutions
Phase 3 KPI	Peer-support network linking pioneer hospitals to newly designated sites established; measurable reduction in time-to-first-use [target to be specified]
Timeline	Operational guidance under the 2024 Regulations within [12] mo; capability program within [18–24] mo
9.3 Redesign the financial model	
Lead (R/A)	Guangdong Provincial Healthcare Security Administration (GDHSA) (proposed lead)
Collaborating (C)	Commercial insurers (e.g., operators of ‘Sui Xin Bao’, ‘Da Ai Wu Jiang’); GDMPA; designated hospitals
Informed (I)	GDHC; patients/public
Legal basis	2024 Guangdong Regulations, Article 22 (encourages commercial health insurance covering HKMEC products)
Phase 1 KPI	GBA-wide inclusive commercial health insurance framework designed (universal catalog coverage; no waiting period; defined reimbursement ratios; dynamic inclusion)
Phase 2 (3-yr) KPI	Insurance coverage for > = 80% of catalog products; measurable reduction in patient out-of-pocket burden
Parallel action	Remove HKMEC drug costs from Guangdong’s annual self-pay ratio benchmark (current rule penalizes high-uptake hospitals)
Timeline	3-year delivery target
9.4 Deepen substantive cross-border medical collaboration	
Lead (R/A)	GDHC, with designated tertiary hospitals (proposed lead)
Collaborating (C)	Hong Kong/Macao medical groups (e.g., HK Greater Bay Area Medical Group); GDMPA; commercial insurers
Informed (I)	NMPA; HK Department of Health; Macao Health Bureau
Model/benchmark	Guangzhou First People’s Hospital Nansha Branch x HK GBA Medical Group (~50% HK/Macao patient representation; ~27% YoY growth)
Phase 1 KPI	Replicable collaboration model extended to 10–15 further HKMEC sites
Phase 2 KPI	Cross-border EHR sharing, HK elder medical voucher acceptance, and commercial-insurance direct payment operational at participating sites
Strategic dependency	Hong Kong’s 2024 Policy Address drug-approval reform; if HK’s reformed process becomes a recognized gateway (EMA/FDA/PMDA-approved drugs) into HKMEC, effective product range expands
Timeline	Site expansion within [24–36] mo [target to be specified]
9.5 Develop a national complementary zoning strategy	
Lead (R/A)	NMPA (policy-level endorsement) (proposed lead)
Collaborating (C)	GDMPA; Hainan PDRA; Lecheng Bureau; NMPA CDE
Informed (I)	Designated institutions of both programs
Strategic framing	HKMEC = large-scale RWD generator for HK/Macao-vetted therapies; Lecheng = first-in-China gateway for globally novel drugs; framed as complementary, not competing
Phase 1 KPI	Formal national ‘complementary zoning’ strategy endorsed at NMPA policy level; coordinated access-and-evidence pipeline defined
Phase 2 KPI	Mutual product-evaluation recognition protocol introduced (approval in one program triggers expedited rather than full review in the other), reducing duplicated expert-panel reviews
Phase 3 KPI	Joint national pharmacovigilance mechanism for cross-system adverse-event data sharing established
Timeline	Policy endorsement within [12–18] mo; mutual-recognition protocol within [24–36] mo [targets to be specified]

All lead entities and KPIs are the authors’ proposals illustrating feasible implementation pathways and do not represent officially mandated responsibilities; designated entities and KPIs would require formal governmental adoption and adjustment. R/A, Responsible/Accountable; C, Consulted; I, Informed. GDMPA, Guangdong Provincial Medical Products Administration; GDHC, Guangdong Provincial Health Commission; GDHSA, Guangdong Provincial Healthcare Security Administration; CDE, Center for Drug Evaluation; PDRA, Provincial Drug Regulatory Authority; EHR, electronic health record; RWD, real-world data; KPI, key performance indicator.

### 9.1 Build a regulation-grade RWD infrastructure

Among the most consequential near-term investments is a regulation-grade RWD platform, built to NMPA standards from inception, with OMOP-CDM compatibility, data dictionaries standardized across all 71 institutions (46), and a federated access architecture enabling cross-center analysis without data leaving institutional systems. Platform governance should rest on a formal three-party cooperation mechanism. This mechanism should be jointly led by the Guangdong Provincial Drug Administration (GDPDA) and the Guangdong Provincial Health Commission (GDHC) and convened with the 71 designated institutions and the NMPA Center for Drug Evaluation (CDE), following the Lecheng model (NMPA CDE–Hainan PDRA–Lecheng Bureau), with legally specified roles, data access protocols, and NMPA submission procedures. A realistic construction horizon of approximately 24 months is achievable, benchmarked against the Lecheng platform (initiated in 2020, operational by 2022). To ensure accountability, implementation should be anchored to measurable milestones: standardized data integration across ≥50% of designated institutions within 24 months, and submission of the first GBA-data-based NMPA registration-support dossier within 36 months (21). Without such governance architecture and delivery discipline, Article 23’s authorization of HKMEC-generated RWE in regulatory submissions risks remaining aspirational (35). Boao Lecheng’s experience proves the effectiveness of standardized platform governance. It adopts a coordinated tripartite mechanism linking NMPA review bodies, provincial regulators and pilot zone authorities, enabling systematic generation of real-world data that can serve as credible evidence for drug registration (49).

### 9.2 Streamline processes and develop institutional capacity

Adopting Lecheng’s “pre-entry, batch-inventory; per-use, batch-clearance” customs model across GBA ports would reduce per-shipment clearance from weeks to days. For catalog-listed products, a notification-and-verification model—under which qualified institutions self-certify compliance and initiate use subject to post-initiation audit—would eliminate duplicative applications. While the 2024 Regulations establish the statutory foundation for this approach, their practical implementation requires detailed operational guidance. Capability development for the 27 fourth-batch institutions is equally urgent. A GDPDRB-coordinated program should provide standardized pharmacy-setup checklists, prescriber training modules, and informed-consent templates. A peer-support network linking pioneer hospitals to newly designated sites would accelerate time-to-first-use.

### 9.3 Redesign the financial model

Universal self-pay pricing in practice constrains HKMEC access primarily to economically advantaged patients, in tension with the program’s equity rationale. The 2024 Guangdong Regulations on HKMEC Drugs and Medical Devices explicitly encourage the exploration of commercial health insurance products covering HKMEC drugs and devices (Article 22), providing a clear legal mandate for payment reform (27). Building on this authorization, the Guangdong Provincial Healthcare Security Administration (GDHSA) should lead the design of a GBA-wide inclusive commercial health insurance framework, calibrated using 5 years of utilization data now increasingly informative for actuarial modeling. The framework should build on the operational experience of Guangzhou’s “Sui Xin Bao” product series (launched March 2026 as the first commercial insurance products in China concurrently covering both HKMEC and provincial/municipal innovative drug catalogs) and Zhuhai’s “Da Ai Wu Jiang,” which pioneered HKMEC inclusion in 2023 (48). It should encompass universal catalog coverage, no waiting period, defined reimbursement ratios reflecting HKMEC price premiums, and dynamic product inclusion. A concrete three-year delivery target should be set: insurance coverage for ≥80% of catalog products, with measurable reductions in patient out-of-pocket burden. In parallel, HKMEC drug costs should be removed from Guangdong’s annual self-pay ratio benchmark. The current rule penalizes designated hospitals for high HKMEC uptake and suppresses prescribing at scale.

### 9.4 Deepen substantive cross-border medical collaboration

The collaboration between Guangzhou First People’s Hospital Nansha Branch and Hong Kong Greater Bay Area Medical Group has achieved approximately 50% Hong Kong/Macao patient representation and around 27% year-on-year growth (34). It establishes a replicable model for substantive clinical integration. Extending this to 10–15 further HKMEC sites, with infrastructure for cross-border electronic health record sharing, Hong Kong elder medical voucher acceptance, and commercial insurance direct payment, would deepen the program beyond drug importation. Hong Kong’s 2024 Policy Address committed to reforming the local drug approval mechanism (50). The reform may create a streamlined pathway for drugs already approved by the European Medicines Agency (EMA), Food and Drug Administration (FDA), or Pharmaceuticals and Medical Devices Agency (PMDA). If Hong Kong’s reformed process becomes a recognized regulatory gateway into HKMEC, the effective product range would expand substantially.

### 9.5 Develop a national complementary zoning strategy

HKMEC and Lecheng are most productively framed as functionally complementary rather than competing programs: HKMEC serves as the large-scale RWD generator for HK/Macao-vetted therapies, while Lecheng serves as the first-in-China gateway for globally novel drugs not yet reaching HK or Macao. A formal national “complementary zoning” strategy, endorsed at the NMPA policy level and integrating both programs within a coordinated access-and-evidence pipeline, would capture their complementary strengths without requiring either to relinquish its comparative advantage. Mutual product evaluation recognition, under which approval in one program triggers an expedited rather than full review in the other, could be introduced alongside a joint national pharmacovigilance mechanism to facilitate the sharing of adverse-event data across both systems. These constitute the most direct and feasible measures for further progress. Both programs have independently approved overlapping products, resulting in duplicated expert-panel reviews. A mutual recognition protocol would help reduce this duplication.

## 10 Discussion

This review traces the five-year trajectory of HKMEC from a regional pilot to a legislatively codified mechanism and documents both its tangible access gains and its persistent structural constraints. Within China’s regulatory landscape, HKMEC occupies an unusual institutional position: it is neither a conventional expedited approval pathway nor a simple cross-border medical arrangement, but a functionally delegated governance model that bridges jurisdictional regulatory gaps under the “one country, two systems” constitutional framework. Anchored in administrative delegation rather than scientific reassessment, this positioning distinguishes HKMEC from most international comparators and defines its analytical value.

The review contributes to the literature in three ways. Firstly, it conceptualizes and substantiates a cross-jurisdictional delegated regulatory governance model for facilitating cross-border drug and device access. Secondly, it shows that a unified, regulation-grade RWD platform is essential for translating early clinical use into evidence usable for downstream regulatory decisions. Thirdly, it underscores that accelerated access must be coupled with diversified financing mechanisms if equity goals are to be met. HKMEC also offers lessons beyond China. It illustrates both the opportunities and the constraints of leveraging external regulatory decisions to accelerate domestic access, particularly where sovereignty considerations, evidentiary standards, and health equity objectives must be balanced concurrently.

### 10.1 International positioning: a cross-jurisdictional delegated model

Previously cited expedited-approval pathways—FDA Accelerated Approval, the EU conditional marketing authorization (CMA), and Japan’s Sakigake designation—operate at the national level and address the speed of bringing a product to market within a single jurisdiction. HKMEC’s defining innovation lies at a different analytical tier: it is a mechanism of cross-jurisdictional delegated regulation, in which central authority is delegated to a province to recognize products already approved by neighboring jurisdictions (Hong Kong and Macao) without standard national registration. The appropriate comparators are therefore cross-border and subnational regulatory-coordination mechanisms. The EU Cross-Border Healthcare Directive (2011/24/EU) coordinates patients’ access to care across member states but does not delegate product-approval authority; Swiss-EU mutual recognition operates between sovereign equals through treaty-based equivalence; ASEAN pharmaceutical harmonization pursues convergence of technical standards rather than delegated approval; and Nordic cross-border prescription recognition addresses dispensing rather than market authorization. Against these, HKMEC is distinctive in operating within a ‘multi-jurisdiction, single-sovereignty’ structure: Hong Kong and Macao retain separate legal and regulatory systems under ‘one country, two systems’, yet share a single sovereign able to delegate approval authority downward to a provincial government—a configuration absent in the inter-sovereign arrangements that characterize the EU, ASEAN, and Nordic frameworks. A systematic comparison across six parameters is provided in Supplementary Table S3. This structural distinctiveness, rather than approval speed, is the locus of HKMEC’s contribution, and it directly conditions the model’s transferability, to which we now turn.

### 10.2 Generalizability: contextual boundaries and transferable design features

The HKMEC model rests on enabling preconditions that are largely specific to its context: central-government delegation of approval authority to a province, the ‘one country, two systems’ status of the SARs as trusted partner jurisdictions under a single sovereign, and a unified provincial platform within a contiguous regional boundary. Several of these are absent in inter-sovereign settings such as the EU or ASEAN, where no single sovereign can delegate approval downward and arrangements instead rely on mutual recognition or harmonization among equals; the model is therefore not transferable in toto. What is portable is a set of discrete design features—delegated recognition of products already approved by a trusted partner, a designated-institution model that contains early-adoption risk, and a real-world-data feedback loop linking front-line use to registration evidence. Accordingly, HKMEC’s relevance to such settings lies in specific design lessons rather than wholesale model transfer; this bounded framing replaces our earlier, unqualified assertion of broad transferability.

We develop these three portable features and their transfer conditions explicitly. First, delegated recognition of products already approved by a trusted partner jurisdiction decouples access speed from full domestic re-review, and is portable wherever a credible reference regulator exists. Second, the designated-institution model concentrates early-adoption risk in a small set of accountable, high-capacity centers, containing safety and liability exposure during cross-border introduction. Third, the real-world-data feedback loop that channels front-line use into registration-grade evidence converts early access into a learning system rather than a one-way exception. These features are separable and can be adopted singly, without replicating HKMEC’s sovereignty structure. A systematic six-parameter comparison of HKMEC against the expedited and cross-border pathways discussed above is provided in Supplementary Table S3.

### 10.3 Limitations and the evidence base

Several limitations should be noted. Our search was restricted to Chinese- and English-language sources, potentially excluding evidence published in Portuguese (relevant for Macao) or other languages. Although we manually searched key regulatory websites, additional gray literature may reside in institutional archives that are not publicly indexed. Evidence published after 30 May 2026 is not included. Given the rapidly evolving policy landscape, new updates may have emerged thereafter. Finally, although we applied a source-tiered appraisal scheme (Methods §2; Supplementary Tables S1, S2), this scheme rates source type rather than internal study quality, so inferences about the methodological rigor of individual outcome studies remain limited.

The evidentiary base of this review is weighted toward T2 government instruments and T3 institutional sources, reflecting the policy-implementation nature of HKMEC and the relative scarcity of peer-reviewed empirical evaluation. This distribution has two implications. First, the regulatory and procedural findings—legal basis, catalog composition, approval mechanism—rest on authoritative primary instruments (T1–T2) and can be stated with confidence. Second, claims bearing on clinical or utilization outcomes draw partly on T3–T4 material whose methodological transparency is limited; these are correspondingly framed as provisional and are not used as standalone evidence of effectiveness. Readers should therefore interpret the policy-architecture findings as well-grounded, while treating outcome-level observations as indicative pending peer-reviewed evaluation. We regard the present predominance of gray and governmental material—rather than independent empirical study—as itself a finding: it signals an evidence gap that future real-world-evidence research on HKMEC should address.

Fundamentally, HKMEC is best understood as an evolving governance arrangement whose long-term contribution will depend on integrating evidence generation, equitable financing, and cross-system collaboration into a coherent regulatory ecosystem.

## Footnotes

1 https://wsjkw.gd.gov.cn/zwyw/content/post_4810167.html, accessed 30 May 2026.

## References

[1] Wu W. China’s health service collaboration in the Guangdong-Hong Kong-Macao Greater Bay Area: barriers and next steps. Front Public Health. (2025) 13:1442328. doi: 10.3389/fpubh.2025.1442328, 40496450 PMC12149214

[2] Yan X Han Z He S. An infrastructural approach to cross-boundary healthcare access: the case of Hong Kong patients in the Greater Bay Area, China. Soc Sci Med. (2026) 390:118891. doi: 10.1016/j.socscimed.2025.118891 41435550

[3] Li H Liu T Wei Y Liang C Liu L Mao Z . Implementation effectiveness and optimization approach of the “Hong Kong and Macao medicine and equipment connect” policy in ensuring drug availability of rare disease. J Pediatr Pharm. (2026) 32:5–9. doi: 10.13407/j.cnki.jpp.1672-108X.2026.04.002

[4] Wang Y Tang Y Du R Long X Yang L. Assessing the “drug loss” and “drug lag” for rare diseases in China: a comparative analysis with the United States (2001-2024). Clin Pharmacol Ther. (2026) 119:1025–35. doi: 10.1002/cpt.70198, 41518196

[5] Han Y Jiang R Li J Wang Y Shao R Xie J. The impact of regulatory reforms in China on drug lag: the role of clinical development strategies. Clin Pharmacol Ther. (2024) 115:1400–7. doi: 10.1002/cpt.3227, 38429944

[6] Liu Y Zhang N Xie C Jiang Y Qin Y Zhou L . Evolution of drug regulations and regulatory innovation for anticancer drugs in China. Acta Pharm Sin B. (2022) 12:4365–77. doi: 10.1016/j.apsb.2022.08.004, 36561995 PMC9764065

[7] Tan R Hua H Zhou S Yang Z Yang C Huang G . Current landscape of innovative drug development and regulatory support in China. Signal Transduct Target Ther. (2025) 10:220. doi: 10.1038/s41392-025-02267-y, 40691459 PMC12280122

[8] Zhu X Xiao D. Innovative drugs approved in China after registration classification reform: current status, disparities, and challenges. Clin Pharmacol Ther. (2026) 119:470–9. doi: 10.1002/cpt.70084, 41075154 PMC12816426

[9] State Administration for Market Regulation (2020) Notice on the GBA drug and medical device regulatory innovation development work plan. Available online at: https://www.beijing.gov.cn/zhengce/zhengcefagui/qtwj/202307/t20230721_3204362.html. (Accessed May 30, 2026)

[10] Lin K. Toward more effective coordination of drug clinical trials in the Greater Bay Area. Asian Rev Polit Econ. (2025) 4:23. doi: 10.1007/s44216-025-00060-w

[11] Lian Y. The “Hong Kong and Macao medicine and equipment connect” (HKMEC): a vivid embodiment of Guangdong’s pioneering spirit. China Health. (2022) 9:83–5. doi: 10.15973/j.cnki.cn11-3708/d.2022.09.009

[12] Huo P Su Z Zheng P. Analysis of the policy implementation process of “Hong Kong and Macao Yao Xie Tong” from the perspective of model of policy implementation system: based on the research of China resources Guangdong pharmaceutical. J Guangdong Pharm Univ. (2022) 38:45–50. doi: 10.16809/j.cnki.2096-3653.2022101106

[13] Xian Z. The Implementation Dilemma and Optimization Path of Medical Policy in Guangdong-HongKong-Macao Greater Bay Area ——a Case Study of the “Hong Kong and Macao Medicine and Equipment Connect” Policy [master’s thesis]. Guangzhou, Guangdong Province, China: Guangdong University of Foreign Studies (2025).

[14] Zhou W Lou X Yang Y Yao Y. Exploring and establishing a real-world data quality management system based on Hong Kong-Macao medicines and devices policy. Chin J Hosp Pharm. (2023) 43:1721–5. doi: 10.13286/j.1001-5213.2023.15.12

[15] Li Y Chen P Wang N. Establishment of evaluation dimensions and indicators for the selection of new drugs based on the “Hong Kong and Macao medicine and equipment connect” policy. China Pharm. (2024) 33:6–9. doi: 10.3969/j.issn.1006-4931.2024.05.002

[16] Cailin B Lou X Li J Cheng G Zhou W. Exploring clinical evaluation tools for drugs under the “Hong Kong and Macao medicine and equipment connect” policy. Pharm Today. (2026) 36:141–6. doi: 10.12048/j.issn.1674-229X.2026.02.010

[17] Akodad S Goldman M Stevens H. Early access disparities in innovative therapies across the US, EU, China, and Japan. Front Med. (2025) 12:1642882. doi: 10.3389/fmed.2025.1642882, 41210884 PMC12588950

[18] Franco P Jain R Rosenkrands-Lange E Hey C Koban MU. Regulatory pathways supporting expedited drug development and approval in ICH member countries. Ther Innov Regul Sci. (2023) 57:484–514. doi: 10.1007/s43441-022-00480-3, 36463352 PMC9734413

[19] Wang S Liu X Zou Y Tang Y Zuo W Jiang R . Trends, lag and characteristics of rare disease drug approval in the USA and China, 1983-2022. Orphanet J Rare Dis. (2025) 20:635. doi: 10.1186/s13023-025-04175-4, 41430297 PMC12750715

[20] Eichler HG Oye K Baird LG Abadie E Brown J Drum CL . Adaptive licensing: taking the next step in the evolution of drug approval. Clin Pharmacol Ther. (2012) 91:426–37. doi: 10.1038/clpt.2011.345, 22336591

[21] Sherman RE Anderson SA Dal Pan GJ Gray GW Gross T Hunter NL . Real-world evidence - what is it and what can it tell us? N Engl J Med. (2016) 375:2293–7. doi: 10.1056/NEJMsb1609216, 27959688

[22] Bootes A Maundu J Golding S McDonald M Lombard J. Fast-track pathways for drug approvals: the Australian experience so far. Aust Prescr. (2019) 42:118–9. doi: 10.18773/austprescr.2019.044, 31427838 PMC6698240

[23] Bloem L Schelhaas J López-Anglada L Herberts C Hennik P Tenhunen O. European conditional marketing authorization in a rapidly evolving treatment landscape: a comprehensive study of anticancer medicinal products in 2006-2020. Clin Pharmacol Ther. (2023) 114:148–60. doi: 10.1002/cpt.2906, 37129347

[24] Xie J Li J Liu Y Wang H Wang Y Yang Y . Comparison of novel oncology drugs that received dual approval from the US accelerated approval and EU conditional marketing authorisation pathways, 2006-2021: a cross-sectional study. BMJ Open. (2023) 13:e069132. doi: 10.1136/bmjopen-2022-069132, 37286329 PMC10255285

[25] Tanaka M Idei M Sakaguchi H Kato R Sato D Sawanobori K . Achievements and challenges of the Sakigake designation system in Japan. Br J Clin Pharmacol. (2021) 87:4027–35. doi: 10.1111/bcp.14807, 33694268

[26] Guangdong Administration for Market Regulation (2021). Circular on issuing the interim provisions for the Administration of Clinically Urgently Needed Imported Hong Kong and Macao Drugs and medical devices in the mainland area of the Guangdong-Hong Kong-Macao Greater Bay Area. Available online at: https://www.gd.gov.cn/zwgk/gongbao/2021/25/content/post_3519273.html. (Accessed May 30, 2026).

[27] Guangdong Provincial People’s Congress (2024) Regulations of Guangdong Province on the Administration of Imported Hong Kong and Macao Drugs and medical devices in the nine mainland cities of the Guangdong-Hong Kong-Macao Greater Bay Area. Available online at: https://www.gdpc.gov.cn/gdrdw/zyfb/ggtz/content/post_199216.html. (Accessed May 30, 2026).

[28] Center for Drug Evaluation, National Medical Products Administration (2021) Guiding principles for real-world data used in generating real-world evidence (trial implementation). Available online at: https://www.cde.org.cn/main/news/viewInfoCommon/2a1c437ed54e7b838a7e86f4ac21c539. (Accessed May 30, 2026).

[29] Guangzhou Medical Association, Guangzhou Hospital Association. Consensus on ethical review of Hong Kong and Macao drug and medical device access in Guangzhou. Guangzhou Med J. (2025) 56:1467–72. doi: 10.20223/j.cnki.1000-8535.2025.11.001

[30] Guangdong Medical Products Administration, Guangdong Health Commission (2026) Notice on issuing the catalogue of clinically urgent imported Hong Kong and Macao drugs and medical devices for the nine mainland cities of the Greater Bay Area (2025 edition). Available online at: http://mpa.gd.gov.cn/ztzl/dwq/sjfb/content/post_4851555.html. (Accessed May 30, 2026).

[31] Sun T Wu Q Yuan Y Zheng Y Fang J. Reflections and suggestions on the current status and optimization directions of the “Hong Kong and Macao medicine and equipment connect” policy. China Food & Drug Administration Magazine. (2025) 8:68–79. doi: 10.3969/j.issn.1673-5390.2025.08.008

[32] Guangdong Medical Products Administration, Guangdong Health Commission (2024) Notice on issuing the catalogue of clinically urgent imported Hong Kong and Macao drugs and medical devices for the nine mainland cities of the Greater Bay Area (2024 edition). Available online at: https://mpa.gd.gov.cn/ztzl/dwq/wjjs/content/post_4607485.html. (Accessed May 30, 2026).

[33] Chen H Zhu H Gao M Cai M Li Y Yang J . Enlightenment of “Hong Kong and Macao medicine and equipment connect” and Lecheng pilot zone policy on anti-tumor drug regulation. Chin Pharm Aff. (2025) 39:528–37. doi: 10.16153/j.1002-7777.2024-12-0043

[34] Lin M Gao W. "Promoting institutional opening-up in the Guangdong-Hong Kong-Macao Greater Bay Area through innovation of the “Hong Kong and Macao medicine and equipment connect” policy". In: Proceedings of the 2024 Emerging Economies Forum. Guangzhou, Guangdong, China: China Society of Emerging Economies, China International Culture Exchange Center, Guangdong University of Technology. (2024). p. 1218–26.

[35] Li P Wang S Chen Y. Use of real-world evidence for drug regulatory decisions in China: current status and future directions. Ther Innov Regul Sci. (2023) 57:1167–79. doi: 10.1007/s43441-023-00555-9, 37624556 PMC10579147

[36] Mohetaer M Tuersun A Li P Wang S Zhang X Chen Y. Drug review and approval policies based on real-world evidence in China and the United States: a comparative study. Clin Ther. (2024) 46:1059–68. doi: 10.1016/j.clinthera.2024.09.009, 39368881

[37] Liu Y Zhu Z. Research on local administrative pilot legislation of the “Hong Kong and Macao medicine and equipment connect”. Rule Law Forum. (2023) 2:151–65.

[38] Center for Drug Evaluation of the National Medical Products Administration (2022) Announcement on issuing the measures for the implementation of real-world drug research jointly issued by the Center for Drug Evaluation of the National Medical Products Administration, Hainan provincial drug administration, and the Administration of Hainan Boao Lecheng International Medical Tourism Pilot Zone. Available online at: https://amr.hainan.gov.cn/himpa/xxgk/tzgg/202211/t20221118_3311237.html. (Accessed May 30, 2026).

[39] Guangdong Provincial Healthcare Security Administration (2024) Notice of the Guangdong provincial healthcare security administration on standardizing medical service Price items for the Guangdong-Hong Kong-Macao medicine and medical device connect policy. Available online at: https://hsa.gd.gov.cn/zwgk/zcfg/ylbz/content/post_4364108.html. (Accessed May 30, 2026).

[40] Yuan J Lu ZK Xiong X Jiang B. Lowering drug prices and enhancing pharmaceutical affordability: an analysis of the national volume-based procurement (NVBP) effect in China. BMJ Glob Health. (2021) 6:e005519. doi: 10.1136/bmjgh-2021-005519, 34518200 PMC8438819

[41] Zhao D Zhou Z. Intended and unintended impacts of ‘4+7’ volume-based drug procurement on the use of drugs in China: a natural experimental study. Healthcare (Basel). (2025) 13:686. doi: 10.3390/healthcare13060686, 40150536 PMC11941950

[42] Wang J Zhang S Wang C Li J Wang R Zhu L. The vacated space of volume/price of the drugs centralized procurement with quantity in secondary and above public hospitals of China. BMC Health Serv Res. (2024) 24:771. doi: 10.1186/s12913-024-11217-3, 38951849 PMC11218069

[43] Health Bureau, Hong Kong Special Administrative Region (2022). The development of primary healthcare in Hong Kong. Available online at: https://www.primaryhealthcare.gov.hk/bp/en/#. (Accessed May 30, 2026).

[44] Wong S Kung K Griffiths S Carthy T Wong M Lo S . Comparison of primary care experiences among adults in general outpatient clinics and private general practice clinics in Hong Kong. BMC Public Health. (2010) 10:397. doi: 10.1186/1471-2458-10-397, 20602806 PMC2908092

[45] Wei X Li H Yang N Wong SY Owolabi O Xu J . Comparing quality of public primary care between Hong Kong and Shanghai using validated patient assessment tools. PLoS One. (2015) 10:e0121269. doi: 10.1371/journal.pone.0121269, 25826616 PMC4380428

[46] Tan H Teo D Lee D Kim C Neo J Sung C . Applying the OMOP common data model to facilitate benefit-risk assessments of medicinal products using real-world data from Singapore and South Korea. Healthc Inform Res. (2022) 28:112–22. doi: 10.4258/hir.2022.28.2.112, 35576979 PMC9117808

[47] Guangdong Center for Adverse Drug Reaction Monitoring (2025) Notice on issuing the work guideline for adverse reaction/event reporting and monitoring of imported Hong Kong and Macao drugs in the nine mainland cities of the Guangdong-Hong Kong-Macao Greater Bay Area and the work guideline for adverse event reporting and monitoring of imported Hong Kong and Macao medical devices in the nine mainland cities of the Guangdong-Hong Kong-Macao Greater Bay Area. Available online at: http://gdadr.gd.gov.cn/gdadr_rule/content/post_4782459.html. (Accessed May 30, 2026).

[48] Fang S Xu J Liu J. Practice and thinking of hospital operation under the policy of “Hong Kong and Macao medical device and pharmaceutical access”: a case study of Nansha hospital, Guangzhou first people’s hospital. Mod Hosp. (2024) 24:1419–22. doi: 10.3969/j.issn.1671-332X.2024.09.029

[49] Cai L Chang L Li Y Zhu M Zhang H. Discuss application of real-world data from the Boao Lecheng pilot zone to support premarket clinical evaluation of medical device. Chinese J Medical Instrumentation. (2023) 47:83–8. doi: 10.3969/j.issn.1671-7104.2023.01.015, 36752013

[50] The Government of the Hong Kong Special Administrative Region (2024) The chief executive’s 2024 policy address: reform for enhancing development and building our future together. Available online at: https://www.policyaddress.gov.hk/2024/en/policy.html. (Accessed May 30, 2026).

[51] Guangdong Medical Products Administration. Special Column on Regulatory Innovation and Development of Drugs and Medical Devices in the Guangdong–Hong Kong–Macao Greater Bay Area. Guangzhou: GDMPA (2026).

[52] The State Council of the People’s Republic of China (2018) Decision of the state council on temporarily adjusting and implementing relevant provisions of the regulations for implementation of the drug administration law of the people’s republic of China in the Hainan Boao Lecheng international medical tourism pilot zone. Available online at: https://www.gov.cn/zhengce/content/2018-12/29/content_5353307.htm. (Accessed May 30, 2026).

[53] Li J Liu L Cao H Yang M Sun X. Use of real-world evidence to support regulatory decisions on medical devices in China and a unique opportunity to gain accelerated approval in “Boao Lecheng pilot zone”. Cost Eff Resour Alloc. (2023) 21:7. doi: 10.1186/s12962-022-00412-w, 36653783 PMC9850538

