# Supplementary material for: Cross-jurisdictional access to innovative drugs and devices: a five-year policy and practice review of the Hong Kong and Macao medicine and equipment connect in the Greater Bay Area of China
Source: Front Med (Lausanne). 2026 Aug 10;13:1897761. doi: 10.3389/fmed.2026.1897761 (PMC13508815; doi:10.3389/fmed.2026.1897761)
Supplement: Supplementary file 1 [file Table_1.docx]

**Supplementary File: Full literature search strategies for all databases**

1. **Differences in Search Terms Between Chinese and English Databases**

Given the differences in controlled vocabularies, field structures, and terminology between Chinese and English databases, differentiated search strategies were adopted:

(1) English databases: A combination of MeSH/Emtree controlled vocabulary and free-text terms in the Title/Abstract fields was applied. Core search terms were: ("Hong Kong and Macao Medicine and Equipment Connect" OR "HKMEC" OR "Hong Kong-Macao Drug and Medical Device Access" OR "Hong Kong/Macao drug importation" OR "cross-border drug access China" OR "GBA Connect" OR "Greater Bay Area" OR "Guangdong-Hong Kong-Macao") AND ("drug regulation" OR "drug approval"[MeSH] OR "real-world evidence" OR "real-world data" OR "cross-jurisdictional approval" OR "expedited access" OR "regulatory pathway"). PubMed used [MeSH Terms] and [Title/Abstract] fields; Embase used Emtree headings combined with the ti, ab, kw fields.

(2) Chinese databases: Keyword-based field matching was applied. CNKI used the "Subject" field; Wanfang and VIP used the "Title + Keywords + Abstract" fields. Core Chinese search terms were: ("港澳药械通" OR "粤港澳大湾区" OR "大湾区药械") AND ("药品监管" OR "真实世界证据" OR "真实世界数据" OR "快速准入" OR "跨境审批" OR "审评审批"). All Chinese-language search strings were translated equivalents of the English terms specified above.

(3) Rationale for differentiated strategies: "Hong Kong and Macao Medicine and Equipment Connect" (港澳药械通) is a policy-specific term unique to China and was therefore included as a restrictive term only in Chinese databases; English searches focused on internationally generalizable methodological literature on "cross-jurisdictional approval" and "expedited access" to preserve sensitivity.

**2. Inclusion Criteria**

Records were eligible for inclusion if they met all the following criteria:

(1) Peer-reviewed studies, governmental policy documents, or industry analytical reports addressing the design, implementation, regulatory framework, or clinical application of the HKMEC policy and related GBA cross-jurisdictional drug-access mechanisms; (2) studies examining real-world data/evidence governance, drug selection, or comparative analyses between HKMEC and other Chinese sub-national pilot programs (e.g., Lecheng); (3) authoritative regulatory documents and official announcements issued by NMPA, the Guangdong Provincial Drug Administration, or other competent authorities relevant to the program.

**3. Exclusion Criteria**

Records were excluded if they met ANY of the following criteria:

(1) Conference abstracts, editorials, commentaries without primary content; (2) duplicate publications (only the most complete or most recent version retained); (3) studies focusing solely on drug clinical efficacy or pharmacoeconomic evaluation without reference to HKMEC or GBA cross-jurisdictional regulatory context; (4) literature not published in Chinese or English; (5) records whose full text could not be retrieved.

**4. Literature screening process**

Records identified from databases (n = 163) and from other sources—government and regulatory websites, policy documents, and media reports (n = 13)—were pooled (n = 176) and imported into reference-management software, where 16 duplicates were removed, leaving 160 records for screening. One reviewer (Mo Zhenjie) conducted the searches, managed the records, and screened titles and abstracts against the eligibility criteria, excluding 37 records at this stage; 123 reports were then sought for retrieval, of which 3 could not be obtained. The remaining 120 reports were assessed against the full eligibility criteria, and 67 were excluded with reasons recorded: not specific to HKMEC (n = 27), no original analysis or data (n = 20), conference abstracts only (n = 8), and redundant policy content (n = 12). A second reviewer (Yuan Yan) independently validated the screening decisions and the final selection, with any discrepancies resolved through discussion. This process yielded 53 sources of evidence for inclusion in the synthesis. The full selection process is summarized in the PRISMA-ScR flow diagram (Supplementary Figure S1).

Screening proceeded in two stages with a pre-specified verification and disagreement-resolution protocol. One reviewer (Mo Zhenjie) screened all titles and abstracts against the predefined eligibility criteria, and a second reviewer (Yuan Yan) independently verified every screening decision across the full record set against the same criteria, flagging any decision with which they disagreed. Full-text assessment of retained records followed the same dual-verification arrangement. Any disagreement was resolved by discussion between the two reviewers against the eligibility criteria until consensus was reached; where consensus could not be reached, a third senior author (Yao Hui) adjudicated. This procedure was applied consistently throughout screening to ensure that no single reviewer's judgment unilaterally determined inclusion.
