# Supplementary material for: Cross-jurisdictional access to innovative drugs and devices: a five-year policy and practice review of the Hong Kong and Macao medicine and equipment connect in the Greater Bay Area of China
Source: Front Med (Lausanne). 2026 Aug 10;13:1897761. doi: 10.3389/fmed.2026.1897761 (PMC13508815; doi:10.3389/fmed.2026.1897761)
Supplement: Supplementary file 2 [file Table_2.docx]

**Supplementary Tables**

*Cross-Jurisdictional Access to Innovative Drugs and Devices: A Five-Year Policy and Practice*

*Review of the Hong Kong and Macao Medicine and Equipment Connect (HKMEC)*

*in the Greater Bay Area of China*

**Supplementary Table S1. Source-tiered evidence appraisal framework**

| **Tier** | **Source type** | **Reliability** | **Permitted use** |
| --- | --- | --- | --- |
| T1 | Peer-reviewed empirical / RWE studies | Highest | Summative & quantitative claims |
| T2 | Official government instruments & gazetted catalogs | High (authoritative primary) | Summative & quantitative claims |
| T3 | Institutional reports, consensus, policy analyses | Moderate | Contextual / interpretive statements |
| T4 | Conference papers, theses, industry / news | Limited | Narrative background only; NOT effectiveness |

*Note. Reliability levels are relative and adapted for policy reviews; GRADE-style certainty ratings are not applied to government instruments. Tier labels (T1–T4) are used consistently in the Methods, in this table, and in Supplementary Table S2.*

**Supplementary Table S2. Tier assignment of all included sources (n = 53)**

| **Ref #** | **Source (short)** | **Type** | **Tier** |
| --- | --- | --- | --- |
| 1 | China’s health service collaboration in the GBA: barriers and next steps | Peer-reviewed journal article | T1 |
| 2 | An infrastructural approach to … China | Journal article / report | T1 |
| 3 | Implementation Effectiveness and Optimization … disease | Peer-reviewed journal article | T1 |
| 4 | Assessing … (NMPA–FDA) | Peer-reviewed journal article | T1 |
| 5 | Impact of Regulatory Reforms in China on Drug Lag | Peer-reviewed journal article | T1 |
| 6 | Evolution of drug regulations for anticancer drugs in China | Peer-reviewed journal article | T1 |
| 7 | Current landscape of innovative drug development & regulatory support in China | Peer-reviewed journal article | T1 |
| 8 | Innovative Drugs Approved in China After Registration Reform | Peer-reviewed journal article | T1 |
| 9 | Notice on the GBA Drug & Medical Device Regulatory Innovation Work Plan (2020 No.159) | Official government instrument | T2 |
| 10 | Toward more effective coordination of drug clinical trials in the GBA | Peer-reviewed journal article | T1 |
| 11 | The HKMEC: A Vivid Embodiment of Guangdong’s Pioneering Spirit | Commentary / institutional | T3 |
| 12 | Analysis of the policy implementation … Guangdong Pharmaceutical | Peer-reviewed journal article | T1 |
| 13 | Xian Z. (2025). The Implementation Dilemma … Policy | Peer-reviewed journal article | T3 |
| 14 | Zhou et al. RWD quality-management system under HK/Macao policy (HKU-SZH) | Peer-reviewed journal article | T1 |
| 15 | Li et al. Evaluation dimensions & indicators for new-drug selection under HKMEC | Peer-reviewed journal article | T1 |
| 16 | Exploring Clinical Evaluation Tools … “Hong Kong and Macao Medicine and Equipment Connect” Policy | Journal article / report | T3 |
| 17 | Early access disparities … across the US, EU, China, and Japan | Peer-reviewed journal article | T1 |
| 18 | Regulatory Pathways Supporting Expedited Drug Development in ICH Countries | Peer-reviewed journal article | T1 |
| 19 | Trends, lag and characteristics of … China, 1983-2022 | Peer-reviewed journal article | T1 |
| 20 | Adaptive licensing: taking the next step in drug approval | Peer-reviewed journal article | T1 |
| 21 | Real-World Evidence: What Is It and What Can It Tell Us | Peer-reviewed journal article | T1 |
| 22 | Fast-track pathways for drug approvals: the Australian experience | Peer-reviewed journal article | T1 |
| 23 | European … Medicinal Products in 2006-2020. | Peer-reviewed journal article | T1 |
| 24 | Comparison of novel oncology drugs … a cross-sectional study. | Peer-reviewed journal article | T1 |
| 25 | Achievements and challenges of the Sakigake designation (Japan) | Peer-reviewed journal article | T1 |
| 26 | Circular on… Clinically Urgently Needed Imported (2021) | Official government instrument | T2 |
| 27 | Regulations of Guangdong Province on … Greater Bay Area | Official government instrument | T2 |
| 28 | Guiding Principles for RWD Used in Generating RWE (Trial) — NMPA | Official government instrument | T2 |
| 29 | Consensus on Ethical Review of HK/Macao Access in Guangzhou | Expert consensus | T3 |
| 30 | Notice on issuing … (2025 edition) | Official government instrument | T2 |
| 31 | Reflections and Suggestions … Policy. | Peer-reviewed journal article | T1 |
| 32 | Notice on issuing … (2024 edition). | Official government instrument | T2 |
| 33 | Enlightenment … on Anti-tumor Drug Regulation. | Journal article | T3 |
| 34 | Promoting institutional … Policy (erenumab single-center case series) | Conference / grey literature | T4 |
| 35 | Use of RWE for Drug Regulatory Decisions in China | Peer-reviewed journal article | T1 |
| 36 | Drug Review & Approval Policies Based on RWE: China vs US | Peer-reviewed journal article | T1 |
| 37 | Research on local administrative pilot legislation of HKMEC | Legislation analysis | T2 |
| 38 | Announcement on Issuing the Measures … Pilot Zone. | Official government instrument | T2 |
| 39 | Notice of the Guangdong Provincial Healthcare … Policy | Official government instrument | T2 |
| 40 | Lowering … China (NVBP / VBP) | Peer-reviewed journal article | T1 |
| 41 | Intended … Study (‘4+7’ procurement) | Peer-reviewed journal article | T1 |
| 42 | The vacated space … China. | Peer-reviewed journal article | T1 |
| 43 | The Development of Primary Healthcare in Hong Kong. | Official government instrument | T2 |
| 44 | Comparison of primary care … Hong Kong | Peer-reviewed journal article | T1 |
| 45 | Comparing quality of public primary care … HK/Shanghai (validated tools) | Peer-reviewed journal article | T1 |
| 46 | Applying the OMOP Common Data Model (Healthc Inform Res) | Peer-reviewed journal article | T1 |
| 47 | Notice on Issuing two GBA HK-Macao imported drug & device adverse-event monitoring guidelines (2025) | Official government instrument | T2 |
| 48 | Practice and Thinking of Hospital Operation under HKMEC: Nansha Hospital case | Journal article / report | T3 |
| 49 | Discuss Application of Real-world Data … Device | Journal article / report | T3 |
| 50 | HK Chief Executive 2024 Policy Address | Official government address | T2 |
| 51 | Special Column on … Greater Bay Area | Official government column / website | T2 |
| 52 | Decision of the State Council on … Law | Official / legal document | T2 |
| 53 | Use of RWE … Pilot Zone | Peer-reviewed journal article | T1 |

*Note. Tier labels follow Supplementary Table S1. Tier distribution: T1 (peer-reviewed / RWE) comprises the majority of academic references, including peer-reviewed Chinese-language journal articles; T2 (government instruments, gazetted catalogs, legislation, policy address): refs 9, 26, 27, 28, 30, 32, 37, 38, 39, 41, 45, 51, 52, 53; T3 (institutional reports, expert consensus, policy analyses, thesis): refs 11, 13, 16, 29, 33, 49, 50; T4 (grey / conference literature): ref 34. Consistent with the downgrade described in the Methods, the erenumab case series (ref 34) is classified T4 and is used for narrative background only, not as effectiveness evidence. Reference numbers correspond to the final manuscript reference list.*

**Supplementary Table S3. Comparison of HKMEC with national expedited-approval and cross-border / subnational regulatory-coordination mechanisms across six parameters**

| **Parameter** | **HKMEC (GBA, China)** | **FDA Accelerated Approval (US)** | **EU CMA** | **Japan Sakigake** | **EU Directive 2011/24/EU** | **Swiss–EU MRA** | **ASEAN harmonization** | **Nordic Rx recognition** |
| --- | --- | --- | --- | --- | --- | --- | --- | --- |
| **Mechanism type** | Cross-jurisdictional delegated regulation | National expedited approval | National conditional approval | National pioneering designation | Cross-border care coordination | Inter-sovereign mutual recognition | Regional standards harmonization | Cross-border dispensing recognition |
| **Jurisdictional architecture** | Multi-jurisdiction, single sovereignty | Single jurisdiction | Supranational (single EU authority) | Single jurisdiction | Multi-state (EU) | Two sovereign parties | Multi-sovereign bloc | Multi-sovereign (Nordic) |
| **What crosses the border** | Approval authority delegated; partner-approved products recognized | Nothing (faster domestic approval) | Nothing (conditional domestic MA) | Nothing (domestic priority) | Patients / reimbursement | Conformity assessments | Technical standards | Prescriptions |
| **Approval / recognition pathway** | Recognize prior HK/Macao approval; no standard NMPA registration | Surrogate endpoints + confirmatory trials | Early MA + post-auth obligations | Priority review + early agency consultation | Care abroad under home-state rules | Re-testing waived via equivalence | Common dossier (ACTD) | Foreign Rx dispensable |
| **Geographic / institutional scope** | Designated institutions, 9 GBA mainland cities | Nationwide | EU-wide | Nationwide | EU patients | CH–EU bilateral | ASEAN members | Nordic states |
| **Post-market surveillance / RWD-to-registration loop** | Yes — traceability platform; RWD feeds later registration | Partial — confirmatory post-approval trials | Partial — specific obligations; annual renewal | No — standard re-examination (~up to 10 yr) | N/A — home-state follow-up | N/A — ongoing conformity | N/A — PV convergence (varies) | N/A — home-state monitoring |

*Note. Only HKMEC simultaneously combines four features absent from every comparator: delegation of approval authority across distinct legal systems, a single-sovereignty structure, recognition of partner-jurisdiction approvals in place of standard domestic registration, and a real-world-data-to-registration feedback loop confined to designated institutions. The three national pathways (FDA Accelerated Approval, EU CMA, Japan Sakigake; refs 18, 23, 25) accelerate review within one jurisdiction and involve no cross-border element; the four cross-border mechanisms (EU Directive 2011/24/EU, Swiss–EU MRA, ASEAN harmonization, Nordic prescription recognition) operate among sovereign equals through mutual recognition or harmonization without delegating approval authority (illustrative comparators, not among the 53 included sources). Abbreviations: HKMEC, Hong Kong and Macao Medicine and Equipment Connect; GBA, Greater Bay Area; CMA, conditional marketing authorization; MA, marketing authorization; MRA, mutual recognition agreement; NMPA, National Medical Products Administration; ACTD, ASEAN Common Technical Dossier; Rx, prescription; RWD, real-world data; PV, pharmacovigilance.*
